# DNA sequence features in the establishing of H3K27ac

**DOI:** 10.12688/f1000research.13441.2

**Published:** 2018-08-09

**Authors:** Anatoliy Zubritskiy, Yulia A. Medvedeva

**Affiliations:** 1 Institute of Bioengineering, Research Center of Biotechnology, Russian Academy of Sciences, Moscow, 119071, Russian Federation; 2 Department of Computational Biology, Vavilov institute of General Genetics, Russian Academy of Sciences, Moscow, 119991, Russian Federation; 3 Department of Biological and Medical Physics, Moscow Institute of Physics and Technology, Dolgoprudny, 141701, Russian Federation

**Keywords:** histone modification, H3K27ac, GC-content, CpG dinucleotides

## Abstract

The presence of H3K27me3 has been demonstrated to correlate with the CpG content. In this work, we tested whether H3K27ac has similar sequence preferences. We performed a translocation of DNA sequences with various properties into a beta-globin locus to control for the local chromatin environment. Our results suggest that in contrast to H3K27me3, H3K27ac gain is unlikely affected by the CpG content of the underlying DNA sequence, while extremely high GC-content might contribute to the gain of the H3K27ac.

## Introduction

Modification of histone proteins is a key mechanism of epigenetic regulation. Histone modifications vary between cells in some genomic locations but not others. This observation raises the question to what extent histone modifications depend on the underlying nucleotide sequences. It has been reported that the attraction of PRC2 complex and consequent H3K27me3 is positively correlated with the local density of CpG dinucleotides ^1^. More complex sequence patterns, such as transcription factor (TF) binding sites (TFBS), also affect the presence of histone modifications. For example, SUZ12, a member of the PRC2 complex, binds DNA in a sequence-specific manner and NRCF and ZBTB33 recruit histone deacetylase. The ENCODE project demonstrated a specific histone modification profile around binding sites of many TFs ^2^.

Since the same lysine residue cannot be both methylated and acetylated, the presence of H3K27ac is negatively correlated with the presence of H3K27me3 ^3^. Although there are several pieces of evidence showing that H3K27me3 is established at least partially in a sequence-specific manner, it is unclear if H3K27ac — an antagonistic activator to a repressive H3K27me3 — shares any sequence specific patterns. A computational approach ^4^ predicted some TFBS to be linked to H3K27ac, yet the results were inconsistent and dependent on the a background set. In this work, we perform a direct experiment to test whether a specific genomic sequence is capable of recovering H3K27ac.

## Methods

### Cas9 target selection and plasmid design

We selected a site for Cas9 in the intergenic region of human beta-globin locus using services CCTop ^5^ (hg38) and Off-Spotter ^6^ (hg38). The chosen targeting sequence was CTTGTCCCTGCAGGGTATTA. Then we designed targeting oligonucleotides ( Table 2) for pSpCas9(BB)-2A-Puro (pX459 plasmid (Addgene), a gift from Egor Prokhorchouck, E.P.). Oligonucleotides Bgl1Cas_F and Bgl1Cas_R were diluted to 10 *µ*M each with annealing buffer (10 mM Tris, pH 7.5, 100 mM NaCl, 1 mM EDTA), heated for 5 minutes to 95°C and then slowly cooled down to room temperature. 500ng of pX459 was digested with 5U of BpiI (Thermo Scientific) for 1h at 37°C, heat inactivated, run on 1.5% agarose, linearized plasmid was cut out and purified with QIAquick Gel extraction kit (Qiagen, cat. no. 28706) and ligated with Bgl1Cas_F/Bgl1Cas_R duplex. The ligation product was transformed into *E. coli* Top-10 cells, one of the clones was chosen and the insert was confirmed by Sanger sequencing. Below we refer to this plasmid as pX459-b1.

### Plasmids design

Selection cassette design (HSV thymidine kinase, T2A peptide, NeoR in one frame) was performed with Ugene ^7^. HSVtk and NeoR sequences were PCR amplified with Phusion polymerase (Thermo Scientific) from pBS246-neo/Tk (a gift from E.P., construction of this plasmid is described elsewhere ^8^) with primers HSVtk_F, HSVtk_R, and G418_F, G418_R, correspondingly. T2A peptide coding sequence, corresponding to amino acid sequence GSGEGRGSLLTCGDVEENPGP, was synthesized by hybridization of oligonucleotides T2A(+) and T2A(-) in annealing buffer and consequent treatment of hybridized duplex with T4 polymerase (Thermo Scientific) and gel purified. The NeoR fragment was digested with XbaI (Thermo Scientific), the T2A fragment was digested with NheI (Thermo Scientific), then these fragments were ligated and the fragment of expected size (881bp) was gel purified. This T2A-NeoR fragment was digested with XhoI (Thermo Scientific), HSVtk fragment was digested with SalI (Thermo Scientific), fragments were ligated and the fragment of predicted length was gel purified again. This HSVtk-T2A-NeoR fragment was double digested with EcoRI (Thermo Scientific) and BshTI (Thermo Scientific) and ligated with pX459-b1 double-digested with EcoRI and BshTI. This step gave the plasmid with full-length selection cassette. Below we refer to this plasmid as pHSVtk-T2A-NeoR. Then the pHSVtk-T2A-NeoR plasmid was double-digested with XbaI and Acc65I (Thermo Scientific), gel purified and ligated with hybridized AdaptUp(+)-AdaptUp(-) duplex. A successful insert was verified by digestion of newly introduced restriction sites: SalI and BamHI. We refer to this plasmid as pAdaptUp-HSVtk-T2A-NeoR. This plasmid was digested with NotI (Thermo Scientific), treated with FastAP (Thermo Scientific), gel purified and ligated with AdaptDown(+)-AdaptDown(-) duplex to introduce a 2xBpiI site that generates half-sites for BclI and XhoI after cleavage. The resulting plasmid was transformed into *E. coli* Top-10 cells. We refer to this plasmid as pAdaptUp-HSVtk-T2A-NeoR-AdaptDown. To obtain LoxP-flanked sequences, we used a pBK-CMV-derived plasmid with a modified multiple cloning site containing restriction sites for BclI, NheI, and XhoI. This plasmid was transformed into *E. coli* JM110 to eradicate Dam methylation, double digested with BclI and NheI, dephosphorylated, gel purified and ligated with LoxP(+)- LoxP(-) duplex, treated with T4 PNK. This plasmid was transformed into *E. coli* Top10 cells and referred to as pLoxP. To obtain homology regions that flank Cas9 cleavage site we PCR amplified them from Caki1 gDNA, using primer pairs Bgl1Up_F and Bgl1Up_R for an upstream fragment and Bgl1 Down_F and Bgl1Down_R for a downstream fragment. These PCR fragments were double digested with NheI and XhoI and ligated with pLoxP, double digested by the same sites and dephosphorylated. Ligation products were transformed into *E. coli* JM110, and purified plasmids were digested with BclI and XhoI to yield upstream and downstream fragments of DNA bearing LoxP site on its end. The upstream fragment was ligated with pAdaptUp-HSVtk-T2A-NeoR-AdaptDown double digested with SalI and BamHI to give pUp1L-HSVTK-T2A-NeoR-AdaptDown. The downstream fragment was ligated with pUp1L-HSVTK-T2A-NeoR-AdaptDown treated with BpiI and dephosphorylated. We refer to this plasmid as pUp1L-HSVTK-T2A-NeoR-L1Down.

### Stably transfected cell line generation

We co-transfected Caki1 cells with the plasmids pX459-b1 and pUp1L-HSVTK-T2A-NeoR-L1Down using Lipofectamine 3000 (Thermo Scientific) in 2cm ^2^ wells following manufacturers instructions. After one week of Puromycin (3 *µ*g/ml) selection, cells were split into 96-well plates and selected with 1mg/ml G418 for two weeks. Cells from successfully growing clones were split into two equal aliquots, one for growth and another for genomic DNA isolation. Clones were checked for the presence of the insert with a primer pair BGL1pcr_F - BGL1pcr_R surrounding the insert. One clone (referred to as Caki1-GcvS-G418R) with a homozygous insert was selected for the further experiments.

### Construction of a recombination target

Plasmid pBK-CMV was digested with SacI and HpaI, blunted with T4 polymerase, self-ligated, transformed into *E. coli* Top10 and called pBCK-CMVdHpaI-SacI. LoxP(+) oligo was PNK treated and annealed with LoxP(-) to form a duplex. Bait(+) and Bait(-) oligos were annealed and ligated with hemi-phosphorylated LoxP duplex. Ligation products were resolved on 3% agarose gel, the longest fragment was excised and purified with QIAquick gel extraction kit, treated with T4 PNK and ligated to NheI treated and dephosphorylated pBCK-CMVdHpaI-SacI. The ligation product was transformed into *E. coli* Top10, we refer to the resulting plasmid as p1L-bait-L1. Ten sequences chosen to be inserted ( Table 1) were PCR amplified from genomic DNA of Caki1 cells with primers ( Table 2, rows 25 to 44). Amplicons were gel purified, diluted to a concentration of 100nM, treated with T4 PNK and ligated with a plasmid p1L-bait-L1 treated with Ecl136II and dephosphorylated. Then the library of ligation products was transformed into *E. coli* Top10, the plasmid library was purified using a plasmid mini kit (Evrogen, cat. no. BC021) and co-transfected with pBS598 EF1alpha-EGFPcre (Addgene) to Caki1-GcvS-G418R cells for Cre-mediated recombination exchange of the insert and the HSVTK-T2A-NeoR cassette. This step was performed in two independent replicates. After 3-day growth Ganciclovir (2 *µ*M) was added to eliminate cells that did not undergo recombination. After selection for 10–14 days survived cells were grown to a subconfluent monolayer in 10cm dishes and then ChIP on H3K27Ac was performed.

**Table 1.** Sequences inserted to foreign genomic context and their properties.

Sample number	Genome location, hg38	Gene name	Length, bp	GC, %	CpGs	H3K27Ac, native location	H3K27ac, foreign location	H3K27Me3, native location
1	chr1:62435851-62436130	USP1	280	59.6	15	yes	no	no
2	chr1:77979434-77979693	FUBP1	260	58	27	yes	no	no
3	chr1:86913854-86914119	HS2ST1	266	56.4	16	yes	no	no
4	chr20:63473349-63473556	-	208	65	1	no	no	yes
**5**	**chr12:49033153-49033412**	**-**	**260**	**66.6**	**1**	**no**	**yes**	**no**
**6**	**chr17:81127213-81127450**	**-**	**238**	**67.7**	**2**	**no**	**yes**	**yes**
7	chr2:227381301-227381590	-	290	26.6	1	no	no	no
8	chr1:108505575-108505854	-	280	37.5	0	no	no	no
9	chr19:50816997-50817284	-	288	51.7	0	yes (only GM12878)	no	yes (in some cells)
10	chrX:48988617-48988801	-	185	52	1	no	no	no

**Table 2.** Oligonucleotides used in this article.

Number	Name	Sequence
1	Bgl1Cas_F	CACCGCTTGTCCCTGCAGGGTATTA
2	Bgl1Cas_R	AAACTAATACCCTGCAGGGACAAGC
3	HSVtk_F	AATTACCGGTATGGCTTCGTACCCCTGCC
4	HSVtk_R	AATTGTCGACGTTAGCCTCCCCCATCTCC
5	T2A(+)	AAAGCTCGAGGGCAGTGGAGAGGGCAGAGGAAGTCTGCTAACATGCGGTG
6	T2A(-)	ATTCGCTAGCTGGGCCAGGATTCTCCTCGACGTCACCGCATGTTAGCAGAC
7	G418R_F	ATTATCTAGAATTGAACAAGATGGATTGCACG
8	G418R_R	TAATGAATTCTCAGAAGAACTCGTCAAGAAGG
9	AdaptUp(+)	CTAGCAGTCGACTTAAGGATCCAT
10	AdaptUp(-)	GTACATGGATCCTTAAGTCGACTG
11	AdaptDown(+)	GGCCGCGATCCTGTCTTCAAGAAGACCTTCGAGC
12	AdaptDown(-)	GGCCGCTCGAAGGTCTTCTTGAAGACAGGATCGC
13	LoxP(+)	CTAGCATAACTTCGTATAATGTATGCTATACGAAGTTATT
14	LoxP(-)	GATCAATAACTTCGTATAGCATACATTATACGAAGTTATG
15	Bgl1Up_F	TCCACTCGAGACCTGGAAACCCATGTCG
16	Bgl1Up_R	GAATGCTAGCTCTGCGTTACACTCTAGTCACAC
17	Bgl1Down_F	CATCGCTAGCCCAGGCATACCAGGCAAATAAG
18	Bgl1_Down_R	TGCTCTCGAGAAATTGACACCATGGCCCAC
19	BGL1pcr_F	TGCTGCAGATACCATCATCC
20	BGL1pcr_R	GTAGAATAGACCTGCACCTGCT
21	Bait(+)	GATCACACTCCTCTGAAGTGAGAGAGCTCCACTAGGACACCTTCTGGT
22	Bait(-)	GATCACCAGAAGGTGTCCTAGTGGAGCTCTCTCACTTCAGAGGAGTGT
23	Bait-seq_F	CACACTCCTCTGAAGTGAGAGAG
24	Bait-seq_R	CCAGAAGGTGTCCTAGTGGAG
25	01_Hi_Ac_FUBP1_F	CCGCTATGGGTCATTCTCGG
26	01_Hi_Ac_FUBP1_R	ATTCAACACCCACCCTCGTG
27	02_Hi_Ac_USP1_F	GGCTGAACGTATCCTGCTTAAC
28	02_Hi_Ac_USP1_R	GCGAATGCTGTAGAGCTGAG
29	03_Hi_Ac_HS2ST1_F	GGGGAAGGAGAAGGATCAACC
30	03_Hi_Ac_HS2ST1_R	TGCGGAAAATGGTAGCGATG
31	04_No_Ac_GC66_F	GGCAGTAGGACCCCCTAGAC
32	04_No_Ac_GC66_R	GCATAACTAAGTCCAGGCCCC
33	05_No_Ac_GC67_F	CTGTGTCCCATAAGGCCCTG
34	05_No_Ac_GC67_R	ACAAACCAAGCTCTGGGTCC
35	06_No_Ac_GC68_F	CCCCCATGGCAAAAGCAAG
36	06_No_Ac_GC68_R	CCCACCACCTTCCAGAGGAG
37	07_No_Ac_GC28_F	CTATCAACACAACACACAAAACACC
38	07_No_Ac_GC28_R	AGGTAAGTAACTACCACCTCCCAC
39	08_No_Ac_GC39_F	AAATCCACTTTTCATCACCACCTC
40	08_No_Ac_GC39_R	TTTAAGAGAAATGGTTAATGTGGGG
41	09_No_Ac_GC51_F	TGAGGTGATTCTGTGGGGTAAC
42	09_No_Ac_GC51_R	ACAACCCCAAAGTCTGGCAC
43	10_No_Ac_GC53_F	TTCAGACACACTCATCTCCCAC
44	10_No_Ac_GC53_R	GGTTAACTGGGTCTGAAGATTCC

### Chromatin immunoprecipitation (ChIP)

ChIP was performed according to Abcam X-chip protocol with the following modification: we increased the number of washes in High Salt buffer from one to three times. Sonication was performed in PCR tubes (SSIbio, cat. no. 3245-00) on ice using Sonics Vibra-Cell VCX 130 with an eight-element probe (cat. no. 630-0602). Sonication setup was: 10s pulse, 20s pause, 75% power, total sonication time is 30min. We used 2 *µ*g of Anti-H3K27Ac antibody (Abcam, ab4729) per ChIP. Biological replicates of targeted inserts of DNA sequences were processed independently. Control ChIP was performed under the same conditions with Caki1 cells.

### Nested PCR

After ChIP, the DNA was pooled and 1ng of DNA was PCR amplified with a primer pair Bait-seq_F – Bait-seq_R. At this step we amplified all DNA sequences inserted between this primer pair. Then 10ng of PCR amplicons from the first step was used as a template for a PCR with a primers ( Table 2, rows 25 to 44), each pair in an individual reaction tube.

## Results and discussion

**Figure 1.**
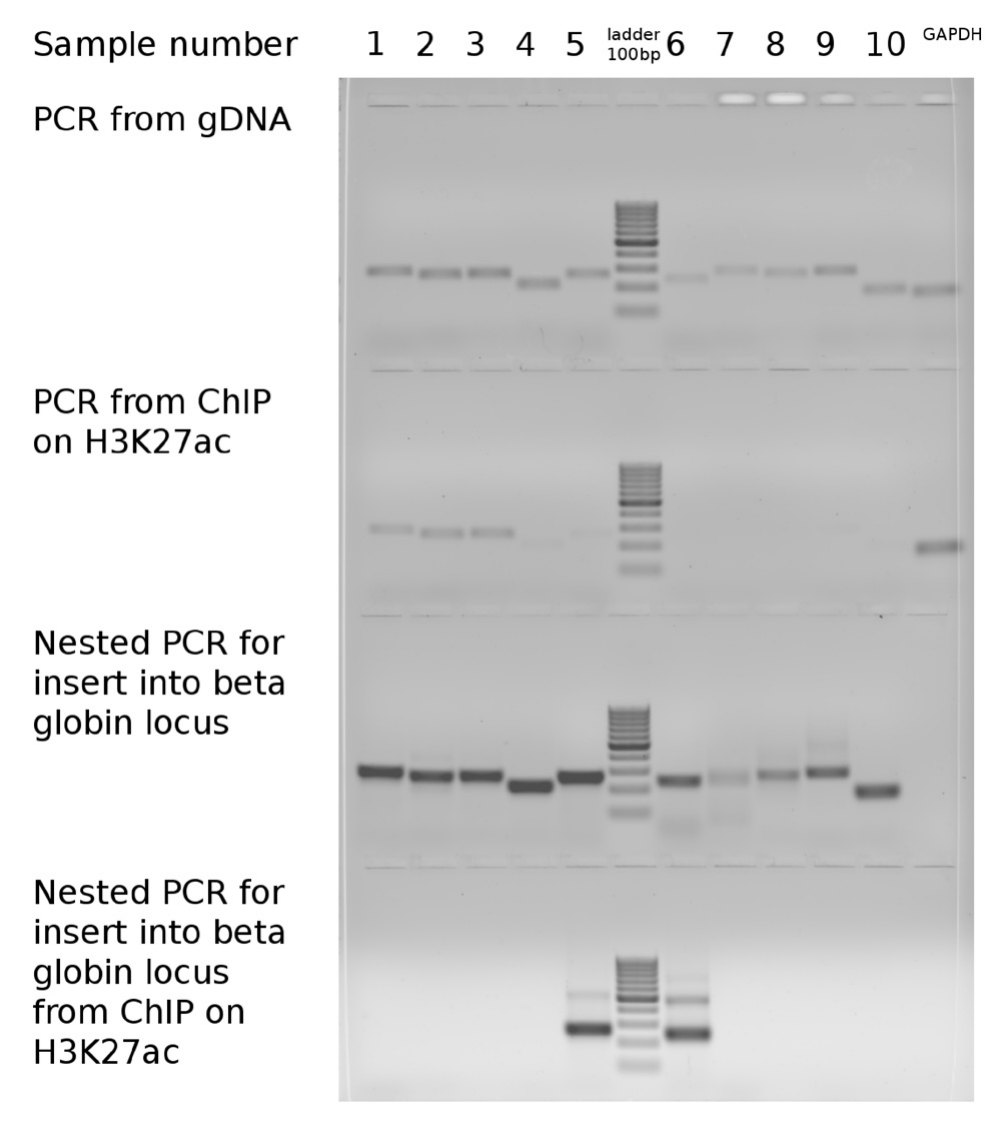
Amplification of chosen sequences in native and foreign genomic context; H3K27ac ChIP-PCR in native and foreign genomic context.

None of the GC- and CpG rich promoter regions, that were acetylated in their original genomic loci (rows 1–3 in Table 1 and lanes 1–3 in Figure 1) recovered H3K27ac after relocation to a foreign genomic context in the beta globin locus, suggesting that H3K27ac may not depend directly on such features. An alternative explanation is that some of the CpG dinucleotides became methylated in a foreign genomic environment preventing chromatin from gaining H3K27ac - an active chromantin mark. Surprisingly, two extremely GC-rich but CpG poor (and, therefore, unmethylated) sequences (rows 5, 6 in Table 1 and lanes 5, 7 in Figure 1) gained H3K27ac in the foreign environment, while in their native environment (in their original genomic location) they had no H3K27ac. Sequences 5 and 6 are located far from promoters of known genes. The sequence 5 contains a lowly expressed CAGE cluster, representing a weak alternative promoter of the KMT2D gene ( ZENBU). Therefore, we conclude that the gain of H3K27ac in these regions is unlikely to be explained by transcriptional activity. The lack of H3K27ac in the sequence 6 in the native location may be due to the presence of the antagonistic mark H3K27me3 in Caki1 cells, which is lost after translocation to a foreign environment. Yet, for the sequence 5, H3K27me3 is absent in all cell types reported in ENCODE. Our results suggest that in contrast to H2K27me3, H3K27ac gain is unlikely affected by the CpG content of the underlying DNA sequence, while extremely high GC-content might contribute to the gain of the H3K27ac.

Gel image of amplification of target sequences from native genomic loci compared with amplification from beta-globin locus with or without ChIP on H3K27ac Click here for additional data file. Copyright: © 2018 Zubritskiy A and Medvedeva YA 2018 Data associated with the article are available under the terms of the Creative Commons Zero "No rights reserved" data waiver (CC0 1.0 Public domain dedication).

## Data availability

The data referenced by this article are under copyright with the following copyright statement: Copyright: © 2018 Zubritskiy A and Medvedeva YA

Data associated with the article are available under the terms of the Creative Commons Zero "No rights reserved" data waiver (CC0 1.0 Public domain dedication). 

Dataset 1: Gel image of amplification of target sequences from native genomic loci compared with amplification from beta-globin locus with or without ChIP on H3K27ac. DOI, 10.5256/f1000research.13441.d192774 ^9^
