# Supplementary material for: DNA sequence features in the establishing of H3K27ac
Source: F1000Res. 2018 Aug 9;7:165. Originally published 2018 Feb 8. [Version 2] doi: 10.12688/f1000research.13441.2 (PMC5989146; doi:10.12688/f1000research.13441.2)
Supplement: Gel image of amplification of target sequences from native genomic loci compared with amplification from beta-globin locus with or without ChIP on H3K27ac [file f1000research-7-17036-s0000.tgz › 99bf15cd-ee7c-48db-93e2-c0aa9a4037ad_Dataset_1.zip!/Dataset 1/Description of samples on gel image.docx]

Description of samples on gel image:

lane number	sample ID	GC content	Genomic coordinates	amplicon length

1	1	59.6%	chr1:62435851-62436130	280bp

2	2	58%	chr1:77979434-77979693	260bp

3	3	56.4%	chr1:86913854-86914119	266bp

4	4	65%	chr20:63473349-63473556	208bp

5	5	66.6%	chr12:49033153-49033412	260bp

6	100bp ladder	NA	NA	100bp - 1000 bp

7	6	67.7%	chr17:81127213-81127450	238bp

8	7	26.6%	chr2:227381301-227381590	290bp

9	8	37.5%	chr1:108505575-108505854	280bp

10	9	51.7%	chr19:50816997-50817284	288bp

11	10	52%	chrX:48988617-48988801	185bp

12	GAPGH	NA	chr12:6534937-6535103	167bp

Row 1:

Samples amplified from genomic DNA of CAKI-1 cells.

Row 2:

Samples amplified from DNA fragments obtained from ChIP of CAKI-1 chromatin with anti-H3K27Ac antibody

Row 3:

Samples amplified in 2nd step of nested PCR from genomic DNA of CAKI-1 cells after insertion of targets 1...10 into beta-globin locus without ChIP.

Row 4:

Samples amplified in 2nd step of nested PCR from DNA fragments obtained from ChIP with anti-H3K27Ac antibody of CAKI-1 cells after insertion of targets 1...10 into beta-globin locus.
