# Supplementary material for: DNA sequence features in the establishing of H3K27ac
Source: F1000Res. 2018 Aug 9;7:165. Originally published 2018 Feb 8. [Version 2] doi: 10.12688/f1000research.13441.2 (PMC5989146; doi:10.12688/f1000research.13441.2)
Supplement: Peer review history

# Peer review: Referee response for version 1

**DOI:** 10.5256/f1000research.14594.r34371

**Published:** 2018-06-04

**Authors:** Oleg Gusev

**Affiliations:** 1 RIKEN, Yokohama City, Kanagawa, Japan

The study is focused on question of relation of primary genomic sequences and H3K27 acetylation preferences. Original method, based on the translocation of the   different CG% and CpG -based sequences to the same genomic locus, followed by  H3K27ac  analysis was employed. The results suggest that that the context is more important than GC or CpG properties of the genomic sequence itself. The results of certain interest for the wide pool of researcher working with epigenetic aspects. At the same time, I believe that involvement of more techniques, such as Chip-Seq would be suggested to confirm the results. Also, it is not clear from the text how many replicates were taken and how general is the conclusion, if, for example, different genomic loci (or different genome, for example extremely GC or AT-rich one)  will be used.

I have read this submission. I believe that I have an appropriate level of expertise to confirm that it is of an acceptable scientific standard.

# Response

**Published:** 2018-07-12

**Authors:** Anatoliy Zubritskiy

**Affiliations:** Research Center of Biotechnology RAS, Russian Federation

Q: The study is focused on question of relation of primary genomic sequences and H3K27 acetylation preferences. Original method, based on the translocation of the   different CG% and CpG -based sequences to the same genomic locus, followed by H3K27ac analysis was employed. The results suggest that that the context is more important than GC or CpG properties of the genomic sequence itself. The results of certain interest for the wide pool of researcher working with epigenetic aspects.

A: We thank the reviewer for a careful reading of the manuscript and useful comments. Although we have to disagree that our results show the context to be more important than GC or CpG content of the sequence. In fact, our results suggest that GC content may contribute to the establishing of H3K27ac. To avoid further confusions we modified the abstract and the results accordingly to make this statement more clear.

“Our results suggest that in contrast to H2K27me3, H3K27ac gain is unlikely affected by the CpG content of the underlying DNA sequence, while extremely high GC-content might contribute to the gain of the H3K27ac”

Q: At the same time, I believe that involvement of more techniques, such as Chip-Seq would be suggested to confirm the results.

A: We agree with the reviewer that a ChIP-seq may provide a more unbiased approach. Yet, a regular ChIP-seq experiment after the insertion seems to produce a lot of waste since only one region is expected to be changed. On the other hand a modified version of ChIP-seq experiment with multiple insertions into the same location may indeed be more unbiased and will hopefully increase the number of tested sequences by the order of magnitude making the conclusions more solid. We are working on this protocol now but we believe that this much more complicated experimental setup is beyond the scope of the current pilot paper.

Q: Also, it is not clear from the text how many replicates were taken and how general is the conclusion, if, for example, different genomic loci (or different genome, for example extremely GC or AT-rich one)  will be used.

A: The insertion of 10 target sequences was performed done in two independent biological replicates, then every replicateate was CHiP'd and resulting DNA was pooled before nested PCR step. To avoid any further confusions the corresponding text was added to the Methods.

Section “Construction of a recombination target”

“This step was performed in two independent replicates.” was added to the

Section “Chromatin Immunoprecipitation (ChIP)”

“Biological replicates of targeted insertion of DNA sequences were processed independently””

Section “Nested PCR”

“the DNA was pooled” was added to section “Nested PCR”

The beta-globin locus was chosen based on its lack of the chromatin modifications in the most of the adult cell types so the surrounding chromatin does not interfere with the inserted sequence and the gain of the modifications in the inserted fragment is determined by its sequence. We agree that additional loci lacking chromatin marks might be also tested, yet for now we focus on the modified ChIP-seq protocol to test many more inserted sequences.

At this stage we are not generalizing our conclusions to other genomes with the extreme AT- or GC-contents of the genome since apart of these changes these organisms may also have a quite different H3K27ac-attracting machinery.

# Peer review: Referee response for version 1

**DOI:** 10.5256/f1000research.14594.r30746

**Published:** 2018-02-20

**Authors:** Vasily N Aushev

**Affiliations:** 1 Department of Environmental Medicine and Public Health, Icahn School of Medicine at Mount Sinai, New York City, NY, USA

This study aimed to test whether H3K27 acetylation depends on the properties of the target sequence itself, or rather defined by the surrounding genomic context. To assess this question, authors translocated sequences with different CG% and CpG content, to the same beta globin locus, and then tested their H3K27ac in the new location.

 As three tested highGC-highCpG  sequences lost their H3K27ac in the new location, authors conclude that the context is more important that GC or CpG properties of the sequence itself. At the same time, two other sequences actually acquire H3K27ac in the same new location, and only one of those can be possibly explained by the antagonistic H3K27me3 mark.

 Authors describe their methods with all the details which should be sufficient for reproducibility of their main results. I had, however, some minor technical questions regarding their plasmids construction procedure. For example: 1) From the sequence of T2A oligos they provide, peptide sequence seems to be PSPLPSPCLLTCGDVEENPGP, not GSGEGRGSLLTCGDVEENPGP. The latter one would be present in the original pX459 plasmid, but not in the constructed one. 2) To my understanding, annealing of T2A(+) and T2A(-) oligos will produce a hybrid with only 18bp double-stranded overlap - for further digestion with NheI, its single-stranded ends need to be built up with some polymerase; I don't see this step in the Methods. 3) I don't see XhoI site in the T2A-NeoR fragment, and don't see any other potential site compatible with SalI of HSVtk fragment. In addition, it is recommended that all the source plasmids used should be properly referred - either by their original publication, or by the sequence reference in any available repository (Genbank, Addgene, etc); referring as "pHSVTK-Neo (a gift from E.P.) " is not sufficient if the plasmid's sequence cannot be explicitly found by that name. Finally, authors should double-check their text for typos in the names of all constructs and genes (for ex., GAPGH), be sure to use all the titles exactly as they are originally defined (for ex., PX459 instead of pX-459, etc). All these issues, however, are minor and do not distort the main content of the paper.

 From the technical point of view, the only question I had is about the final readout for H3K27ac: authors perform nested PCR followed by visualization of the PCR products on the gel. I am wondering this method was chosen and not qPCR (as more quantitative) or ChIP-seq (as more unbiased).

 One of my main concerns is about the reproducibility of the findings: authors do not report any biological or technical replicates which makes the results potentially questionable.

 Talking about the interpretation of the results, I am not sure how strong can be the conclusion based on only 10 tested sequences, and even more so, only one genomic context. Even with this limited number of tests, some results cannot be easily explained (for example, acquired H3K27ac for sample #5), so in future it would be necessary to expand the panel of both sequences and contexts.

 Besides above-mentioned questions (hopefully authors can address at least some of them in the revised version), I think the paper is clearly of scientific interest and adds important piece of knowledge to its field.

I have read this submission. I believe that I have an appropriate level of expertise to confirm that it is of an acceptable scientific standard.

# Response

**Published:** 2018-07-12

**Authors:** Anatoliy Zubritskiy

**Affiliations:** Research Center of Biotechnology RAS, Russian Federation

We would like to thank the reviewer for the thorough reading of our paper and very useful comments.

Q: From the sequence of T2A oligos they provide, peptide sequence seems to be PSPLPSPCLLTCGDVEENPGP, not GSGEGRGSLLTCGDVEENPGP. The latter one would be present in the original pX459 plasmid, but not in the constructed one.

A: We have to politely disagree with the reviewer on this point. According to https://eu.idtdna.com/calc/analyzer in hetero-dimer mode, the annealed T2A(+)/T2A(-) duplex with minimal dG value looks as follows:

AAAGCTCGAGGGCAGTGGAGAGGGCAGAGGAAGTCTGCTAACATGCGGTG

                               ||||||||||||||||||                  

                               CAGACGATTGTACGCCACTGCAGCTCCTCTTAGGACCGGGTCGATCGCTTA

For a double-stranded DNA synthesized with the T4 polymerase, the (+) strand looks as follows:

AAAG **CTCGAG**GGCAGTGGAGAGGGCAGAGGAAGTCTGCTAACATGCGGTGACGTCGAGGAGAATCCTGGCCCA **GCTAGC**GAAT

This sequence contains two restriction sites (XhoI and NheI). The sequence between restriction sites is translated as GSGEGRGSLLTCGDVEENPGP in 5’->3’ direction, frame 1 ( https://web.expasy.org/cgi-bin/translate/dna2aa.cgi).

Q: To my understanding, annealing of T2A(+) and T2A(-) oligos will produce a hybrid with only 18bp double-stranded overlap - for further digestion with NheI, its single-stranded ends need to be built up with some polymerase; I don't see this step in the Methods.

A: We thank the reviewer for pointing out this mistake. The typo in Methods section is corrected and enzyme name is changed from T4 PNK to T4 Polymerase. We modified the Methods section and added the following text:

"T2A peptide coding sequence corresponding to amino acid sequence GSGEGRGSLLTCGDVEENPGP was synthesized by hybridization of oligonucleotides T2A(+) and T2A(-) in annealing buffer and consequent treatment of hybridized duplex with T4 polymerase (Thermo Scientific) followed by gel purification."

Q: I don't see XhoI site in the T2A-NeoR fragment, and don't see any other potential site compatible with SalI of HSVtk fragment.

A: The T2A oligonucleotide duplex contains an XhoI site ( CTCGAG) in a 5’ region

AAAG **CTCGAG**GGCAGTGGAGAGGGCAGAGGAAGTCTGCTAACATGCGGTGACGTCGAGGAGAATCCTGGCCCAGCTAGCGAAT

and a primer HSVtk_R contains a SalI restriction site ( GTCGAC) AATT **GTCGAC**GTTAGCCTCCCCCATCTCC compatible with the XhoI site.

Q: In addition, it is recommended that all the source plasmids used should be properly referred - either by their original publication, or by the sequence reference in any available repository (Genbank, Addgene, etc); referring as "pHSVTK-Neo (a gift from E.P.) " is not sufficient if the plasmid's sequence cannot be explicitly found by that name. Finally, authors should double-check their text for typos in the names of all constructs and genes (for ex., GAPGH), be sure to use all the titles exactly as they are originally defined (for ex., PX459 instead of pX-459, etc). All these issues, however, are minor and do not distort the main content of the paper.

A: We carefully checked all the names of the genes and plasmids as well as the rest of the text for typos. The plasmid name pHSVTK-Neo is internal; this plasmid is refered as pBS246-neo/tk in the folowing paper: https://www.ncbi.nlm.nih.gov/pmc/articles/PMC1317619/. The reference describing a the plasmid pBS246-neo/tk is added to the main text:

"HSVtk and NeoR sequences were PCR amplified with Phusion polymerase (Thermo Scientific) from plasmid pBS246-neo/Tk (a gift from E.P.), construction of this plasmid is described elsewhere: https://www.ncbi.nlm.nih.gov/pmc/articles/PMC1317619/"

Q: From the technical point of view, the only question I had is about the final readout for H3K27ac: authors perform nested PCR followed by visualization of the PCR products on the gel. I am wondering this method was chosen and not qPCR (as more quantitative) or ChIP-seq (as more unbiased).

A: For a pilot study we decided to focus on making sure that the insertion of the selected fragments into a foreign environment (beta-globin locus) did take place as a proof of principle therefore we have chosen to perform nested PCR. In this regards qPCR will not make the conclusions more reliable. Performing a regular ChIP-seq experiment after the insertion seems like a bit of overkill since histone mark only in one region is expected to be changed. On the other hand a modified version of ChIP-seq experiment with multiple insertions may indeed be more unbiased. Yet, we believe that this much more complicated experimental setup is beyond the scope of the current paper.

Q: One of my main concerns is about the reproducibility of the findings: authors do not report any biological or technical replicates which makes the results potentially questionable.

A: The insertion of 10 target sequences was performed in two independent biological replicates, then every replicate was ChIP'd independently and resulting DNA was pooled before nested PCR step. To avoid any further confusions the corresponding text was added to the Methods:

Section “Construction of a recombination target”

“This step was performed in two independent replicates.” was added to the

Section “Chromatin Immuniprecipitation (ChIP)”

“Biological replicates of targeted insertion of DNA sequences were processed independently”

Section “Nested PCR”

“the DNA was pooled”

Q: Talking about the interpretation of the results, I am not sure how strong can be the conclusion based on only 10 tested sequences, and even more so, only one genomic context. Even with this limited number of tests, some results cannot be easily explained (for example, acquired H3K27ac for sample #5), so in future it would be necessary to expand the panel of both sequences and contexts.

A: We agree with the reviewer that some of the results (seq #5) are puzzling although reproducible since we performed all the tests in two biological replicates. At this stage we decided to report the results of the pilot project and focus on the performing a more complex experiment based on ChIP-seq as mentioned above which hopefully will increase the number of tested sequences by the order of magnitude.

Q: Besides above-mentioned questions (hopefully authors can address at least some of them in the revised version), I think the paper is clearly of scientific interest and adds important piece of knowledge to its field.

A: Again, we are grateful to the reviewer for the high evaluation of our work, careful reading of the manuscript and useful comments.
